# Supplementary material for: The interactome of intact mitochondria by cross-linking mass spectrometry provides evidence for coexisting respiratory supercomplexes
Source: Mol Cell Proteomics. 2017 Dec 8;17(2):216–32. doi: 10.1074/mcp.RA117.000470 (PMC5795388; doi:10.1074/mcp.RA117.000470)
Supplement: Supplemental Data [file supp_RA117.000470_133922_0_supp_23978_fzffwf.zip › spectra_annotation/mito_DR_spectra_annotation/162-1-11-1-7-1.pdf]

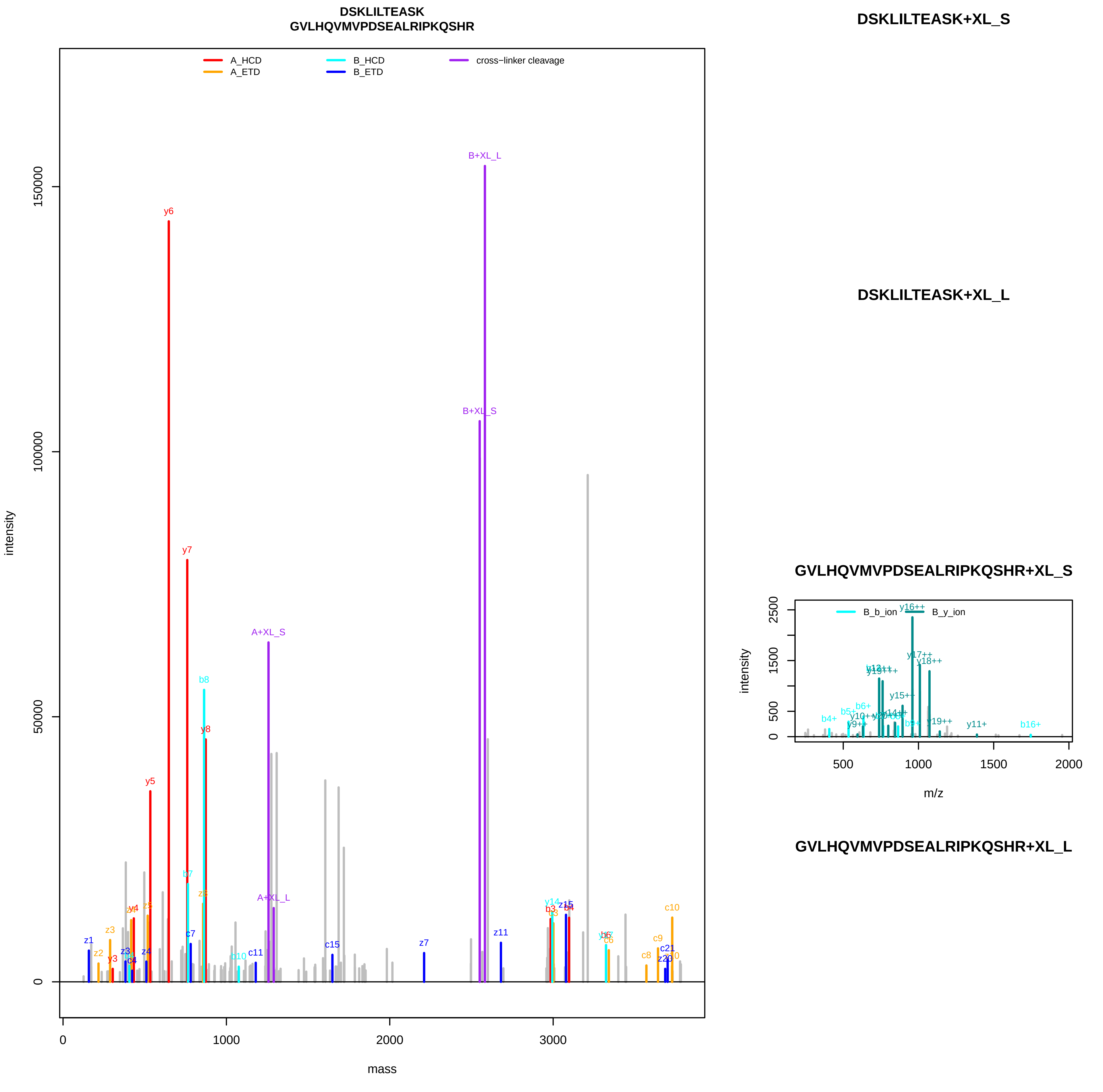
DSKLILTEASK
GVLHQVMVPDSEALRIPKQSHR
A_HCD
A_ETD
B_HCD
B_ETD
cross−linker cleavage
intensity
mass
B+XL_L
B+XL_S
A+XL_S
A+XL_L
y6
y7
y8
y5
b8
b7
y4
y3
z1
z2
z3
z4
c4
c7
c11
c15
z7
z11
c8
c9
c10
c21
z20
DSKLILTEASK+XL_S
DSKLILTEASK+XL_L
GVLHQVMVPDSEALRIPKQSHR+XL_S
B_b_ion
B_y_ion
y16++
y17++
y18++
y15++
b4+
b5+
b6+
y11+
b16+
y19++
m/z
GVLHQVMVPDSEALRIPKQSHR+XL_L
